# Occurrence and Health Risk Assessment of Cadmium Accumulation in Three *Tricholoma* Mushroom Species Collected from Wild Habitats of Central and Coastal Croatia

**DOI:** 10.3390/jof8070685

**Published:** 2022-06-29

**Authors:** Ivan Širić, Pankaj Kumar, Ebrahem M. Eid, Archana Bachheti, Ivica Kos, Dalibor Bedeković, Boro Mioč, Miha Humar

**Affiliations:** 1 Faculty of Agriculture, University of Zagreb, Svetosimunska 25, 10000 Zagreb, Croatia; isiric@agr.hr (I.Š.); ikos@agr.hr (I.K.); dbedekovic@agr.hr (D.B.); bmioc@agr.hr (B.M.); 2 Agro-Ecology and Pollution Research Laboratory, Department of Zoology and Environmental Science, Gurukula Kangri (Deemed to Be University), Haridwar 249404, Uttarakhand, India; 3 Biology Department, College of Science, King Khalid University, Abha 61321, Saudi Arabia; ebrahem.eid@sci.kfs.edu.eg; 4 Botany Department, Faculty of Science, Kafrelsheikh University, Kafr El-Sheikh 33516, Egypt; 5 Department of Environmental Science, Graphic Era (Deemed to be University), Dehradun 248002, Uttarakhand, India; bachheti.archana@gmail.com; 6 Department of Wood Science and Technology, Biotechnical Faculty, University of Ljubljana, Jamnikarjeva 101, 1000 Ljubljana, Slovenia; miha.humar@bf.uni-lj.si

**Keywords:** cadmium, health risk assessment, heavy metals, health hazard, mushrooms, *Tricholoma* spp.

## Abstract

This study deals with the biomonitoring of cadmium (Cd) heavy metal in the three selected *Tricholoma* mushroom species collected from wild habitats of central and coastal Croatia. For this, mushroom (*T. columbetta*: *n* = 38, *T. portentosum*: *n* = 35, and *T. terreum*: *n* = 34) and surface soil samples were collected from nine forest localities of Croatia and analyzed for Cd concentration using inductively coupled plasma–optical emission spectrometry (ICP–OES) through the acid digestion method. The findings revealed that Cd was present in *Tricholoma* spp. and surface soil. However, the maximum mean Cd concentration (mg/kg dry weight) was recorded in *T. portentosum* (cap: 0.98; stipe: 0.72), followed by *T. columbetta* (cap: 0.96; stipe: 0.73) and *T. terreum* (cap: 0.81; stipe: 0.63). The bioconcentration factor (BCF) value (>1) revealed that the selected *Tricholoma* spp. had the potential for Cd accumulation. Moreover, the principal component (PC) and hierarchical cluster (HC) analyses were used to derive the interactions and similarities between Cd levels *Tricholoma* spp. and sampling localities. The multivariate analysis suggested that central sampling localities had higher Cd levels as compared to coastal localities. However, the daily intake of metals (DIM < 0.426) and health risk index (HRI < 1) showed that there was no potential health risk associated with the consumption of selected *Tricholoma* spp. The findings of this study are helpful to understand the Cd accumulation behavior of wild edible *Tricholoma* spp. collected from Croatia.

## 1. Introduction

Around the globe, population growth, intensive industrialization, and urbanization have led to environmental pollution, especially in soil and water. The anthropogenic disposal of pollutants in the environment, especially heavy metals, has become an unavoidable problem affecting different life forms. Cadmium (Cd) is a toxic metal found mostly in trace amounts in the Earth’s crust, with an average concentration of 0.36 mg/kg in soils [1]. The presence of Cd in the soil is a consequence of natural processes and anthropogenic practices. In the natural pedogenetic processes, the soil takes up heavy metals from the parent substrate, whereas in the anthropogenic processes, various activities, such as urbanization, industrialization, trade, and agricultural production, lead to heavy metal mixing in environmental areas. The geogenic origin of Cd is usually associated with sulfur minerals, which oxidizes relatively quickly in the environment, and the metal cation separates from sulfur at an early stage of mineral degradation. In the later stages of pedogenesis, Cd is more common in the composition of Mn oxide [2]. Anthropogenic sources of Cd in the environment include atmospheric deposition, industrial and municipal waste discharge, phosphate fertilizers, pesticides, sewage sludge, ores, metal industry, mining, and incidents [1,3]. Cadmium is considered a very dangerous pollutant to the ecosystem, and, unlike other plants in the terrestrial environment, mushrooms effectively absorb it from the soil [4,5]. Cadmium is a carcinogenic element that adversely affects the kidneys, bones, cardiovascular system, and immune system and belongs to Group 1, according to the International Agency for Research on Cancer (IARC) classification [6]. Anthropogenic sources of Cd pollution have been very important causes of its deposition in forest soils in recent decades. Although everyday knowledge of the toxicity of Cd on the environment and human health has led to its reduction in use in some areas. Still, anthropogenic sources continue to increase in certain areas, which negatively affects the natural landscapes, including forests where mushrooms mainly grow.

Mushrooms belong to the kingdom of fungi and are classified as a distinct microbiological group of organisms of significant nutritional, pharmaceutical, and ecological value. Wild edible mushrooms are considered high-quality foods due to their natural nutritional benefits, including aromatic and antioxidant properties [7,8]. They are a good source of nutritionally important elements, such as K, Zn, Cu, and Mn [9] and B-group vitamins, vitamin D, proteins, and dietary fiber [10]. Additionally, many species of mushrooms are used as medicines to prevent diseases, such as hypertension [11] and hypercholesterolemia [12], and to improve the immune system [13]. Additionally, mushrooms play an important role in the ecosystem because they can degrade many complex molecules of plants and animals [9]. In a symbiotic relationship, the mushroom benefits from plants’ easy access to food. Similarly, the plant benefits because the mushroom produces mycelia, which aids in the absorption of water and nutrients. However, it is known that mushrooms can accumulate certain heavy metals (essential and toxic) and develop their fruiting bodies under conditions that are toxic to most other organisms [14]. Mushrooms can absorb certain forms of heavy metals, such as Cd^2+^, Cd^6+^ Hg^2+^, As^5+^, etc., in their fruiting bodies. In regard to this, intracellular speciation and uptake of metals are generally regulated by metallothioneins and GT-complexes that are directly connected to fungal physiology. Previous studies suggest that mushrooms have an effective system that allows them to absorb heavy metals in a form that does not affect their growth and development. Due to the extremely efficient system of absorption and storage of heavy metals, mushrooms have an extremely good bioaccumulation potential, which depends on many external (environmental) factors and internal mechanisms within the fungus [15]. Thus, various environmental factors, such as the type of soil, the content of organic matter in the soil, nitrogen, the pH value, and the concentration of metals in the soil, as well as the species of fungus or the morphological part of the fruiting body (cap and stalk), the fructification time, age of mycelium, and production of ligands, can influence the accumulation of heavy metals in mushrooms [16,17,18,19,20,21]. Biological and molecular mechanisms of heavy metal uptake in mushrooms have been presented by [22]. The authors state that mushrooms can accumulate metals by passive and active uptake mechanisms. Through a passive uptake mechanism, heavy metals are trapped in the cell structure and then adsorbed to binding sites. The active mechanism of metal uptake is carried out by the biological metabolic process of heavy metal transfer in the cell through the cell membrane [23]. Additionally, according to Mleczek et al. [24], the organic acids secreted by the mushrooms can chelate poorly soluble mineral components of the soil, facilitating and accelerating their uptake by the hyphae and their accumulation in the fruiting body of mushrooms.

Cadmium concentration and its distribution in different mushroom species has been studied by numerous authors around the world [9,14,15,17,25,26,27,28,29,30]. Additionally, the potential toxic effect of Cd from fungi was determined by Sarikurkcu et al. [31]. A wide range of Cd concentrations was found in the edible mushroom species, with concentrations in uncontaminated areas in the range of <0.5 to 2 mg/kg dm, while concentrations in contaminated areas were as high as 10 mg/kg dm [9,14,15,17,25,26,27,28,29,30,32]. Thus, high concentrations of Cd in mushroom edible parts may have adverse effects on human health. Some other studied species that can accumulate Cd are: *Agaricus bisporus* [33,34,35]; *A. campestris* [17,26,36]; *A. macrosporus* [37]; *Armillaria mellea* [30,38,39]; *Amanita muscaria* and *A. allies* [19]; *Boletus edulis* [32,36,40,41,42,43,44]; *Cantharellus cibarius* [40,45,46,47,48]; *Cystoderma carcharias* [49]; *Macrolepiota procera* [25,26,36,50,51]; and *Xerocomus badius* [24,30,36,41,52]. The concentration of Cd in mushroom species of the genus *Tricholoma* usually varies in the range of <0.5 to 1 mg/kg [17,26,30,36,53]. *Tricholoma* spp., belonging to the class Agaricomycetes and genus *Tricholoma*, are found throughout the world, but they are most common in temperate and subtropical climates in both the southern and northern hemispheres. *Tricholoma* spp. are distinguished by hyaline, subglobose to oblong spores, simple pileipellis structures, and a lack of well-differentiated sterile elements, including cystidia [54]. Some *Tricholoma* spp., such as *T. matsutake*, are characterized by a high accumulation of Cd (48.52 mg/kg dm) [29], therefore making it crucial to monitor the Cd content present in commonly consumed *Tricholoma* spp. in Croatia. Studies on the monitoring of Cd contamination of *Tricholoma* spp. are lacking, particularly in central and coastal Croatia. Therefore, keeping in mind the concerns regarding Cd occurrence, this paper aimed to (i) determine the Cd concentration in the *Tricholoma* spp. and its soil substrate; (ii) compare the distribution of the Cd in anatomical parts, i.e., cap and stipe; (iii) determine the suitability of the studied mushroom species as Cd bioaccumulators; and (iv) study the potential health risks associated with the consumption of Cd contaminated mushrooms of the genus *Tricholoma*.

## 2. Materials and Methods

### 2.1. Mushrooms and Forest soil Sampling

**Figure 1:**
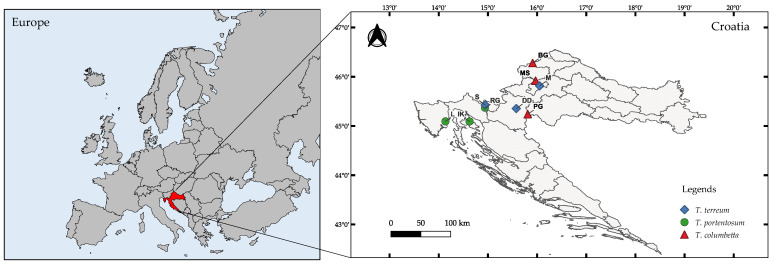
Map of the *Tricholoma* spp. mushroom sample collection sites across Croatia.

The sampling of mushrooms of the *Tricholoma* spp. and substrates (soil) was carried out in nine localities in Croatia, of which five localities were in the central zone and four in the coastal zone (Table 1 and Figure 1). The sampled localities are interspersed with mixed forests of deciduous and coniferous trees. A total of 107 samples of three mushroom species (*Tricholoma columbetta*: *n* = 38, *T. portentosum*: *n* = 35, and *T. terreum*: *n* = 34) were collected from July 2012 to November 2014. Fully developed and mature fruiting bodies of mushrooms were collected by random selection from two large regions in Croatia. At the same time, samples of topsoil (0–10 cm) were collected at mushroom sampling sites (*n* = 177; 10 samples for each site) using the quadrate sampling method [55]. After their collection, the mushroom bodies were thoroughly washed and cut into two anatomical parts, i.e., the cap (pileus) and the stipe (stipes), using a sterile knife, followed by drying at 60 °C to achieve a constant weight. The drying of the mushroom samples was performed in a food and plant dryer (MSG-01; MPM Product, Milanówek, Poland, and Ultra FD1000 dehydrator, Ezidri, Australia). After drying, the samples were ground in a laboratory mill (Retsch SM 200) and passed through a 1.0 mm diameter sieve, followed by storage in air-tight plastic bags until further Cd analysis. Similarly, the forest soil samples were also dried at room temperature, ground using a laboratory mill and passed through a 1.0 mm pore size sieve.

### 2.2. Analysis of Cadmium

The concentration of Cd was determined using inductively coupled plasma–optical emission spectroscopy (ICP–OES). Validation of the method for Cd content was performed using certified reference material (IAEA-336) lichens. The reported Cd concentration for the reference material was 0.117 mg/kg. The recovery result for Cd in this study was 0.120 ± 0.003 mg/kg (*n* = 3), which showed good agreement with the certified levels. Detection limits of Cd were 0.003 mg/kg. The laboratory glassware used to prepare samples for the determination of Cd was soaked in a solution of ethylenediaminetetraacetic acid (EDTA) at a concentration of 5% (*w*/*v*) for 24 h and then in 10% (*v*/*v*) HNO_3_ for 24 h. A total of 0.5 g dried mushroom sample was digested with 5 mL of HNO_3_ (65%, Suprapur, Merck, Darmstadt, Germany) in closed PTFE vessels inside a microwave destruction oven (Milestone Microwave Laboratory System, MLS 1200 mega, Shelton, CT, USA). The destruction program consisted of several steps, such as at a power of 100 W and duration of 5 min; at a power of 0 W and duration of 2 min (“standby time”); at a power of 250 W and duration of 5 min 20; at a power of 400 W and duration of 5 min; and at a power of 600 W and duration of 5 min. After destruction in the microwave, the samples were cooled in a water bath and transferred via a funnel into 25 mL plastic volumetric flasks. After that, the flasks were filled with distilled water. The soil samples were filtered through filter paper (Sigma-Aldrich, St. Louis, MO, USA). From the volumetric flasks, the samples were transferred to plastic tubes for measurement with ICP-OES (Optima 8000, Perkin Elmer, Waltham, MA, USA) equipped with an autosampler, by which Cd concentration was analyzed.

### 2.3. Bioconcentration and Health Risk Index (HRI) Calculation

Bioaccumulation by living organisms is expressed as the tendency of accumulating a specific quantity of heavy metals from their growing environment [35]. The bioconcentration factor (BCF) values were calculated as the ratio between the concentration of Cd in mushroom samples and the forest soil. Furthermore, the health risk index (HRI) was calculated according to Chui et al. [56] by using Equation (1):

HRI = DIC/RfD(1)

According to the above equation, DIC represents daily Cd intake from the consumption of the analyzed mushroom species, while RfD represents the extent of exposure to oral contaminants during life and is mainly used in health assessments [57]. The following Equation (2) was used to calculate the daily intake of Cd [29,56]:

DIC = SM × MCM/ABW(2)

where SM—serving of mushroom (0.03 kg of dried mushrooms), MCM—Cd concentrations in mushrooms (mg/kg dry weight), and ABW—average body weight (70 kg for a regular consumer). In the calculations of the health risk index (HRI), the values of the daily consumption of dried mushrooms (30 g) and the bodyweight of a regular consumer (70 kg) were assumed [45].

### 2.4. Data Analysis and Statistics

All samples were analyzed in three replicates. Descriptive data analysis included minimum value, maximum value, median, and mean and standard deviation (SD) were calculated using the Statistica 10.0 (Statsoft, Tulsa, OK, USA). The map of the study area was generated using QGIS (Version 3.22.3-Białowieza, Open Source, Gispo Ltd., Helsinki, Finland) software, while the principal component and cluster analyses were performed using the OriginPro (Version 2022b, OriginLab, Northampton, MA, USA) software packages.

## 3. Results and Discussion

### 3.1. Cadium Contents in Tricholoma spp. Collected from Central and Coastal Croatia

**Figure 2:**
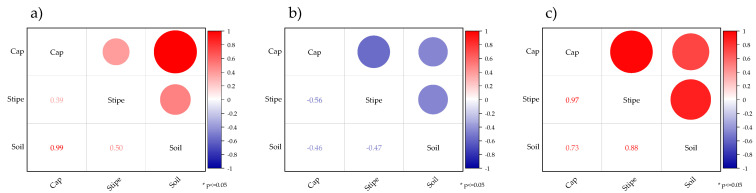
Correlation between Cd contents in the soil, cap, and stipe parts of *Tricholoma* spp. ((**a**) *T. columbetta*, (**b**) *T. portentosum*, and (**c**) *T. terreum*); * indicates level of significance (*p* < 0.05); refer to the color scale bar for interpretation of correlation coefficient values as text and circles.

In the current study, Cd contents in the analyzed *Tricholoma* spp. and forest topsoil are listed in Table 2. The results showed considerable differences in the content of Cd accumulated by three *Tricholoma* spp. across the sampling locations. Nevertheless, the differences in Cd content in forest soils from different sampling localities were also observed. The ICP–OES analysis revealed that Cd contents were present in both the cap and stipe regions of *Tricholoma* spp. at all locations of sample collection. Specifically, the samples of *T. columbetta* mushroom collected from Brezova Gora and *T. portentosum* from Labinstina showed identical Cd concentrations, i.e., 0.91 ± 0.13 and 0.89 ± 0.16 mg/kg, respectively. However, the concentration of Cd in the forest topsoil was relatively low, ranging between 0.07 and 0.57 mg/kg in Brezova Gora, with mean values lying within the range from 0.17 ± 0.03 mg/kg (Ravna Gora) to 0.28 ± 0.09 mg/kg (Stubaki). However, the Cd concentrations in *T. columbetta* and forest soil samples collected from the central region were almost identical to those in the species *T. portentosum* and associated soil, some of whose samples were collected at sites on the Croatian coastal locations. Regarding this, the highest Cd was found in the cap samples of *T. portentosum* at Ravna Gora (0.99 mg/kg), while the lowest average Cd content was found in the stem samples of *T. terreum* at Skrad (0.59 mg/kg). Moreover, the average Cd analysis in the full body of *Tricholoma* spp. showed that *T. columbetta* had the highest concentration, followed by *T. portentosum* and *T. terreum*. Overall, the analyzed samples of the *T. terreum* showed relatively lower Cd content as compared to *T. columbetta* and *T. potrentosum*. Figure 2a–c shows the correlation between Cd contents in soil and cap and stipe parts of three *Tricholoma* spp.

The Cd levels in the investigated *Tricholoma* spp. mushrooms in this study are comparable to the results previously reported by other authors [26,27,36,58,59]. Comparatively, the average Cd concentrations determined in the forest topsoil of Ravna Gora (0.17 ± 0.07 mg/kg dm) region were analogous to those reported in the Szczecinek (0.17 ± 0.06 mg/kg) area of Poland [60] and Yunnan province (0.17 ± 0.03 mg/kg dm) of China [27]. In their study, Petkovšek and Pokorny [14] noted elevated levels of Cd in forest soils can be caused by pollution from nearby anthropogenic sources, such as industrial processes, smelters, and agricultural production. Similar levels of Cd (0.91 mg/kg) in *T. argyraceum* are also reported by Soylak et al. [58]. In addition, Saba et al. [61] reported similar results of Cd (0.91 mg/kg) in *Suillus gavillei* ectomycorrhizal mushroom species. Similarly, *T. terreum* samples collected from a Mediterranean region of Turkey showed average Cd values of 4.90 mg/kg [31], which is considerably higher than the results established in this study. On the other hand, Severoglu et al. [25] found very low Cd levels in the *T. terreum* samples collected in the central region of Turkey (0.05 mg/kg dm).

### 3.2. Bioconcentration Factor (BCF) of Cd Accumulation in Tricholoma spp.

In this study, the bioconcentration factor (BCF) values were calculated to estimate the Cd accumulation potential of selected *Tricholoma* spp. From the upper layer of forest soil (Table 2). The determined values of the BCF indicate whether Cd is actively bioaccumulated (BCF > 1) or not (BCF < 1) by selected *Tricholoma* spp. The BCF values for cap parts of *Tricholoma* spp. Were considerably higher as compared to those for stipes. The highest reported median BCF value was 5.85 for the *T. portentosum* in Ravna Gora with a mean value of 6.17 ± 1.77. Similarly, the specified BCF median value for *T. portentosum* was 3.16 times higher than in the case of *T. columbetta* in Medvednica, Stubaki, where medium BCF was only 1.85. Moreover, the BCF values established for *T. portentosum* in Ravna Gora, also indicated a potential for Cd accumulation. It is well known that mushrooms of the genus *Tricholoma* have a good Cd accumulation potential. Since Cd contamination in the upper layer of soil may be triggered by several anthropogenic activities, thus, higher BCF values were found for some sapling locations in this study. Therefore, the central Croatian sampling locations reported relatively higher BCF values as compared to those in coastal locations. Overall, the BCF values for *T. portentosum* in the Ravna Gora showed the highest Cd bioavailability of all the sampling locations. The concept of BCF is widely accepted by the scientific community for determining the hazardous metal accumulation by edible mushrooms. In a report by Širić et al. [21], the BCF values of Hg metal accumulated by four *Tricholoma* spp., such as *T. equestre*, *T. portentosum*, *T. columbetta*, and *T. terreum*, were observed between 18 to 37 in southern and northern regions of Europe. Similarly, Kojta et al. [60] also reported a BCF value > 40 for Cd accumulation by the *Macrolepiota procera* saprophytic mushroom in the Augustowska forest region of Poland, respectively.

### 3.3. PCA and HCA Results

**Figure 3:**
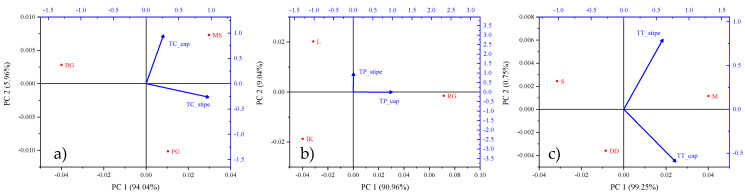
PCA biplot showing interactions between sampling locations and Cd contents in the cap and stipe parts of *Tricholoma* spp. ((**a**) *T. columbetta*, (**b**) *T. portentosum*, and (**c**) *T. terreum*).

**Figure 4:**
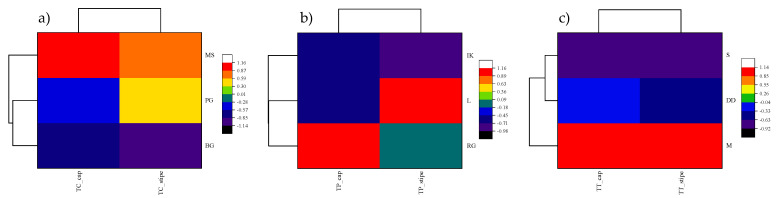
Clustered heatmap showing similarities between sampling locations and Cd contents in the cap and stipe of *Tricholoma* spp. ((**a**) and TC: *T. columbetta*, (**b**) and TP: *T. portentosum*, (**c**) and TT: *T. terreum*).

Principal component analysis (PCA) is a widely accepted statistical tool for deriving the interactive effects of multiple variables based on their dominance [35]. In the current study, the data of Cd concentration in three *Tricholoma* spp. samples were analyzed using PCA based on their collection locations in central and coastal Croatia. In the case of *T. columbetta*, the data were orthogonally transformed onto two principal components, i.e., PC1 and PC2, with variances of 94.04 and 5.96%, respectively (Table 3). Regarding this, the highest concentration of Cd in *T. columbetta* was suggested in the stiped parts at the Petrova Gora (PG) site. However, the highest Cd contents in the cap parts of *T. columbetta* were observed at the Medvednica location as indicated by the vector length of the biplot axis (Figure 3a). Similarly, the PCA-based chemometric assessment of Cd contents in the *T. portentosum* mushroom collected from three coastal zones of Croatia revealed that the two extracted PCs had variances of 90.96% (PC1) and 9.04% (PC2). Contradictorily, maximum Cd levels were observed in the cap parts of *T. portentosum* mushroom collected from RG location as revealed by its vector length dominance in the PC1 data group (Figure 3b). Similarly, PCA results of Cd contents in *T. terreum* mushroom samples collected from two central zones and one coastal zone showed that the cap part indicated the highest concentration at central zone locations (Maksimir and Dugi Dol) (Figure 3c). The percentage of variance distribution among the two PCs was identified as 99.25% (PC1) and 0.75% (PC2). This theorizes that soils of central Croatian zones were more responsible for high Cd uptake by selected *Tricholoma* spp. Hence, the PCA tool was helpful to relate the effect of central and coastal Croatian sampling locations with Cd contents in *Tricholoma* spp. On the other hand, the similarities between sampling locations and Cd levels in *Tricholoma* spp. samples were evaluated using the hierarchical cluster analysis. As depicted in Figure 4a–c, it was observed that Brezova Gora and Medvednica locations showed the highest similarities in terms of Cd contents analyzed in *T. columbetta*; however, Petrova Gora showed a slight similarity, which might be because all three locations are within central Croatia. On the other hand, the Cd contents in T. portentosum mushroom showed no significant difference amongst the three sampling locations, viz., Ravna Gora, Island Krk, and Labinština. However, notable similarities were seen in the case of Cd levels in *T. terreum* mushrooms at the Skrad and Dugi Dol sampling sites. Regarding this, the Maksimir site showed high variation for Cd levels in *T. terreum* mushroom. Previously, Kumar et al. [34] used PCA and HCA approaches to derive the interrelationship between heavy metal levels in *Agaricus bisporus* and their sampling locations across the thirteen districts of Uttarakhand State in India. They revealed that PCA and HCA were useful to understanding the impact of sampling location on the availability of eight heavy metals, including Cd in *A. bisporus* samples. Similarly, Buruleanu et al. [62] also used the PCA tool to study the effect of heavy metal concentration on different biochemical constituents of wild and cultivated mushroom species in Romania. The results of these reports are in line with the current study and suggest that effective information can be derived from the multivariate analysis of Cd level data in *Tricholoma* spp. samples collected from central and coastal Croatian locations.

### 3.4. Health Risk Assessment of Cd Intake

In this study, the potential risk of Cd intake from the consumption of wild edible mushrooms *Tricholoma* spp. was established by using the provisional tolerable daily intake value PTDI (0.5 μg/kg bw/d) for a person of 70 kg body weight [63]. Based on the determined Cd concentrations in mushrooms and the assumed meal (300 g fresh or 30 dried mushrooms per day) [45], the daily intake of Cd (DIC) was calculated as given in Table 4. Here, the range of DIC values in the cap part of *Tricholoma* spp. was 0.329–0.426, while 0.254–0.312 for the stiped. The results showed that the highest DIC was determined in caps of *T. portentosum* at Ravna Gora (0.426 μg/kg body weight/serving). On the other hand, the highest DIC for Cd (0.312 μg/kg body weight/serving) was found in stipes of *T. columbetta*. However, the Skrad sampling location showed the lowest (0.254 g/kg body weight/serving) DIC values. In the case of the health risk index (HRI), the highest value was encountered in the case of *T. portentosum* (0.852) at the Ravna Gora location for the cap parts. For the stiped parts, the highest HRI values (0.624) were observed at the Medvednica, Stubaki, location in *T. columbetta*. Overall, the determined HRI values were below 1 for Cd levels in all analyzed samples of *Tricholoma* spp. in both central and coastal Croatia (Table 4). However, Leung et al. [64] stated that established health risk index values of 1 or less are considered safe for human health.

With the increasing number of wild mushrooms consumers, it has become a topic of great importance to biomonitoring the presence of toxic elements and their potential risks [61]. Regarding this, the elevated Cd levels in wild edible mushrooms can harm consumers’ health, particularly as several species of *Tricholoma* mushroom are consumed in fresh or processed form. However, the practice of monitoring Cd levels before consuming these wild mushrooms is almost entirely lacking in Croatia. Being classified as a “probable” human carcinogen (IARC), the health risk assessment in the current study suggests an association between Cd exposure and the occurrence of cancer in humans [65]. There are several methods of preparing wild edible mushrooms, particularly *Tricholoma*, the most common of which are heat-treated, dried, or pickled. Maintaining regular physiological functions in the human body requires a diet with the optimal intake of essential elements (Fe, Zn, Cu, Mn, Mo, Se) [66], and their deficiency or excessive intake can cause health problems [67]. In addition to essential elements, there are also non-essential elements (Al, As, Ba, Cd, Hg, Ni, Pb) that have no biological functions in the body and are considered dangerous/toxic to consumers [66]. In regard to this, Cd is a well-known food contaminant possessing destructive health effects. Therefore, the toxicological effects of Cd associated with food consumption, in this case of mushrooms whose samples have HRI values > 1, may pose a health risk. The results presented in the current study are consistent with those reported in previous studies. Recently, Sarikurkcu et al. [31] and Chen et al. [27] found an HRI value for Cd greater than 1 in *T. terreum* and *T. matsutake* species. Similarly, Barea-Sepúlveda et al. [68] also calculated HRI values > 1 for Cd in the ectomycorrhizal mushroom species *A. caesarea*, whose samples were collected in Spain and Morocco. Thus, the HRI tool in the present study was helpful for biomonitoring the health risk associated with intake of Cd-contaminated *Tricholoma* spp.

## 4. Conclusions

This study investigated the occurrence of Cd metal in three *Tricholoma* mushroom species (*T. columbetta*, *T. portentosum*, and *T. terreum*) and their adjoining soil substrates across central and coastal Croatia. Results revealed that the highest Cd contents were observed in the *T. portentosum* mushroom followed by *T. columbetta* and *T. terreum*. However, the bioconcentration factor values revealed that selected *Tricholoma* spp. are good Cd accumulators and could uptake considerable amounts of Cd into their vegetative parts from soils. Overall, the health risk studies suggested no potential health risk associated with the consumption of *Tricholoma* spp. According to this study, exposure to Cd through the consumption of contaminated *Tricholoma* spp. is unlikely to cause adverse human health effects if the health risk index (HRI) value goes above 1. Furthermore, continuous monitoring of other toxic heavy metals in wild edible mushrooms in other regions of Croatia is highly recommended.

## Figures and Tables

**Table 1:** Description of study sites of *Tricholoma* spp. mushroom sample collection.

Site Name and Sample Size	Code	Longitude (N)	Latitude €	Zone Type	*Tricholoma* spp.
Brezova Gora (*n* = 14)	BG	15.909140	46.281183	Central Croatia	*T. columbetta* (*n* = 38)
Medvednica, Stubaki (*n* = 14)	MS	15.969287	45.919902	Central Croatia	
Petrova Gora (*n* = 10)	PG	15.810489	45.239646	Central Croatia	
Ravna Gora (*n* = 11)	RG	14.940796	45.369653	Coastal Croatia	*T. portentosum* (*n* = 34)
Island Krk (*n* = 10)	IK	14.626754	45.090944	Coastal Croatia	
Labinština (*n* = 13)	L	14.135917	45.093335	Coastal Croatia	
Maksimir (*n* = 10)	M	16.052633	45.814058	Central Croatia	*T. terreum* (*n* = 35)
Dugi Dol, Karlovac (*n* = 12)	DD	15.576698	45.354304	Central Croatia	
Skrad (*n* = 13)	S	14.947011	45.425098	Coastal Croatia	

**Table 2:** Cadmium contents in *Tricholoma* spp. and related forest soil (mg/kg dry weight), Q_C/S_ index, and BCF values (mean ± SD, median, and range).

Mushroom Species, Localities, and Number of Specimens	Cd Concentration				BCF_cap_	BCF_stipe_	BCF_fullbody_	Q_C/S_
	Cap	Stipe	Full Body	Soil				
*T. columbetta*, Brezova Gora, *n* = 14	0.91 ± 0.13	0.66 ± 0.06	0.78 ± 0.16	0.24 ± 0.14	5.44 ± 4.07	3.95 ± 2.93	5.04 ± 4.39	1.38 ± 0.12
	0.90	0.65	0.75	0.23	3.89	2.84	3.26	1.40
	(0.75–1.11)	(0.57–0.78)	(0.57–1.10)	(0.07–0.57)	(1.37–14.84)	(1.09–10.19)	(1.93–8.14)	(1.18–1.60)
*T. columbetta* Medvednica, Stubaki, *n* = 14	0.94 ± 0.11	0.73 ± 0.06	0.83 ± 0.14	0.38 ± 0.09	2.67 ± 0.86	2.08 ± 0.75	2.67 ± 0.70	1.29 ± 0.14
	0.96	0.73	0.80	0.37	2.43	1.85	2.16	1.27
	(0.76–1.13)	(0.61–0.83)	(0.60–1.13)	(0.19–0.52)	(1.54–5.07)	(1.32–4.19)	(2.17–3.16)	(1.10–1.60)
*T. columbetta* Petrova Gora, *n* = 10	0.92 ± 0.11	0.71 ± 0.05	0.81 ± 0.13	0.23 ± 0.08	4.54 ± 1.85	3.46 ± 1.06	4.49 ± 1.75	1.29 ± 0.15
	0.88	0.72	0.79	0.21	4.23	3.40	3.76	1.23
	(0.81–1.17)	(0.64–0.77)	(0.63–1.17)	(0.11–0.36)	(2.45–9.06)	(2.13–5.90)	(3.25–5.73)	(1.15–1.54)
*T. portentosum* Island Krk, *n* = 10	0.88 ± 0.06	0.66 ± 0.02	0.83 ± 0.17	0.27 ± 0.08	3.67 ± 1.85	2.78 ± 1.32	4.03 ± 1.60	1.33 ± 0.09
	0.90	0.67	0.80	0.30	3.00	2.27	2.67	1.35
	(0.78–0.94)	(0.63–0.69)	(0.62–1.13)	(0.12–0.39)	(2.34–8.56)	(1.61–6.19)	(2.90–5.17)	(1.18–1.45)
*T. portentosum* Ravna Gora, *n* = 11	0.99 ± 0.08	0.68 ± 0.03	0.77 ± 0.11	0.17 ± 0.04	6.17 ± 1.77	4.20 ± 1.04	5.19 ± 1.56	1.46 ± 0.11
	0.98	0.68	0.73	0.17	5.85	3.99	4.29	1.46
	(0.88–1.13)	(0.63–0.72)	(0.63–0.94)	(0.10–0.23)	(4.35–9.36)	(3.11–6.14)	(4.09–6.30)	(1.32–1.59)
*T. portentosum* Labinština, *n* = 13	0.89 ± 0.16	0.70 ± 0.08	0.79 ± 0.15	0.21 ± 0.05	4.59 ± 1.57	3.60 ± 1.11	4.48 ± 1.12	1.26 ± 0.14
	0.87	0.72	0.76	0.20	4.14	3.49	3.80	1.23
	(0.69–1.19)	(0.59–0.81)	(0.58–1.18)	(0.11–0.32)	(2.63–7.92)	(2.31–6.52)	(3.69–5.27)	(1.06–1.57)
*T. terreum* Maksimir, *n* = 10	0.80 ± 0.05	0.64 ± 0.01	0.71 ± 0.09	0.30 ± 0.09	2.78 ± 1.06	2.18 ± 0.76	2.94 ± 1.23	1.27 ± 0.06
	0.81	0.63	0.69	0.33	1.91	1.91	2.09	1.28
	(0.73–0.87)	(0.62–0.66)	(0.61–0.87)	(0.16–0.42)	(1.86–5.43)	(1.57–4.03)	(2.07–3.81)	(1.17–1.35)
*T. terreum* Dugi Dol, Karlovac, *n* = 12	0.77 ± 0.04	0.60 ± 0.02	0.68 ± 0.09	0.18 ± 0.07	4.64 ± 1.59	3.67 ± 1.26	4.28 ± 2.01	1.28 ± 0.09
	0.77	0.59	0.66	0.17	4.53	3.69	3.88	1.28
	(0.68–0.83)	(0.58–0.65)	(0.57–0.83)	(0.10–0.29)	(2.82–8.25)	(2.05–5.79)	(2.86–5.70)	(1.13–1.43)
*T. terreum* Skrad, *n* = 13	0.75 ± 0.03	0.59 ± 0.02	0.66 ± 0.08	0.23 ± 0.09	3.61 ± 1.36	2.85 ± 1.02	3.55 ± 1.69	1.26 ± 0.07
	0.74	0.58	0.66	0.22	3.43	2.56	3.00	1.26
	(0.68–0.81)	(0.57–0.65)	(0.57–0.80)	(0.12–0.34)	(2.16–5.93)	(1.79–4.54)	(2.35–4.75)	(1.14–1.39)

Q_C/S_ (cap to stipe Cd content quotient); BCF_cap_, BCF_stipe_, and BCF_fullbody_ (bioconcentration factor values for caps, stipes, and full body).

**Table 3:** PCA matrix for Cd concentration in cap and stipe parts of *Tricholoma* spp.

Mushroom Species	Variables	Principal Component	
		PC 1	PC 2
*T. columbetta* (*n* = 38)	Variance (%)	94.04	5.95
	Eigenvalues	0.0013	0.0003
	Cd cap	0.99	−0.01
	Cd stipe	0.01	0.99
*T. portentosum* (*n* = 34)	Variance (%)	90.96	9.04
	Eigenvalues	0.0038	0.0001
	Cd cap	0.99	−0.01
	Cd stipe	0.01	0.99
*T. terreum* (*n* = 35)	Variance (%)	99.25	0.75
	Eigenvalues	0.0013	0.0005
	Cd cap	0.79	−0.60
	Cd stipe	0.60	0.80

**Table 4:** Daily intakes of Cd and health risk index in wild edible *Tricholoma* spp. mushrooms.

Species	Locations	Daily Intakes of Cd (DIC, μg/kg Body Weight/Serving)		Health Risks Index (HRI)	
		Cap	Stipe	Cap	Stipe
*T. columbetta*	Brezova Gora	0.388	0.281	0.776	0.562
	Medvednica, Stubaki	0.403	0.312	0.806	0.624
	Petrova Gora,	0.393	0.306	0.787	0.612
*T. portentosum*	Island Krk	0.379	0.284	0.757	0.569
	Ravna Gora	0.426	0.292	0.852	0.585
	Labinština	0.382	0.301	0.764	0.602
*T. terreum*	Maksimir	0.334	0.272	0.689	0.544
	Dugi Dol, Karlovac	0.329	0.258	0.658	0.516
	Skrad	0.320	0.254	0.639	0.508

## Data Availability

Not applicable.
